# Synthesis of Al–Al_2_O_3_–CNF Composite by Cold Spray Method: Powder Preparation and Synthesized Objects Characterization

**DOI:** 10.3390/nano12091559

**Published:** 2022-05-04

**Authors:** Anton Yu. Nalivaiko, Vitaliy V. Doroshenko, Nguyen Kuang, Dmitriy Yu. Ozherelkov, Ivan A. Pelevin, Alexander A. Gromov

**Affiliations:** 1 MISIS Catalysis Lab, National University of Science and Technology MISIS, 119991 Moscow, Russia; v.doroshenko@mail.ru (V.V.D.); nquang.misis@mail.ru (N.K.); d.ozherelkov@gmail.com (D.Y.O.); i.pelevin@misis.ru (I.A.P.); a.gromov@misis.ru (A.A.G.); 2 Directorate of Science, Moscow Polytechnic University, 107023 Moscow, Russia

**Keywords:** additive manufacturing, aluminum, alumina, carbon nanomaterials, cold spray method

## Abstract

This paper is devoted to studying the composite material of the aluminum–alumina–carbon nanofiber (CNF) system. The paper considers in detail the process of preparation of the specified composite by ball milling, as well as the process of synthesis of a solid object (coating) by the cold spray method. The synthesized objects were studied using optical and electron microscopy, and the hardness of objects of various compositions was measured. The processes of interaction of composite particles are discussed in detail. The influence of CNF on the distribution of particles in a solid object and on the hardness of objects has been considered and discussed.

## 1. Introduction

Aluminum and its alloys are widely used in aerospace, automotive, and other fields due to their advantages such as high specific strength and rigidity, low density, and excellent heat and electrical conductivity [1,2,3,4]. However, low hardness limits the use of aluminum in complex engineering applications, which led to the development of aluminum-based composites to improve strength and hardness [5,6]. An alternative approach to increasing the hardness of aluminum alloys is surface treatment technologies. Currently, there are many ways to improve the surface properties of materials, for example, laser weld deposition [7,8], thermal spraying [9,10], anodizing [11,12], and others. Among the methods considered, the cold spray method is a relatively new and perspective manufacturing process [13,14]. During the synthesis of coatings by thermal methods, operating temperatures reach or exceed the melting point of metal particles, which leads to their melting, compression after cooling, and, as a result, the occurrence of residual stress. When using the cold spray method, the particles do not reach the melting point and form a solid material due to the high kinetic energy of collision [15]. Moreover, unlike traditional thermal spray processes, in cold spray coating synthesis, the particles remain in the solid phase due to the relatively cold temperature of the working gas [16]. Thus, the cold spray method can be used to modify the surface of various metallic parts, increasing their mechanical, corrosion, and friction properties [17,18]. In particular, it is applied to aluminum alloys to form strong bonding between surface and material without causing undesirable effects such as surface oxidation, material melting, phase transformations, and changes in the chemical composition at the interface [19]. In previous studies [20,21,22], the process of aluminum–alumina coatings forming by the cold spray method was considered in detail. These studies were aimed at investigating the morphology of the initial particles’ dependence on the performance properties of synthesized coatings/solid objects. In particular, it was found that alumina in the composition of the source material significantly increases wear resistance, hardness, and adhesive strength [23,24]. Moreover, good adhesion and strong bonding between Al_2_O_3_ and aluminum substrate were found, especially when some Al was added to the feedstock powder for cold-spray [18].

In paper [25], the cold spray synthesis of aluminum–carbon nanotube (CNT) coatings was studied. Within that study, several compositions of composite materials containing from 0.2 to 4% CNTs were considered. The synthesized alumina with 2% CNT composite showed the best wear resistance and hardness, wherein, CNTs acted in two ways: (a) as a strengthener of composite surface and (b) made a self-lubricating surface improving friction properties.

In the study [26], a combined method for the formation of aluminum–carbon nanotube coatings was considered, including the cold spray method and plasma electrolytic oxidation. A composite consisting of aluminum and 1% CNT was investigated, and an increase in the coating hardness was found. It is known [27,28,29,30] that the modification of Al-containing composites with carbon nanomaterials has a positive effect on the properties of the obtained objects and leads to an increase in mechanical properties and improves functional characteristics.

The most important carbon nanoscale modifiers are CNT [31] and carbon nanofibers (CNF) [32] due to their exceptional mechanical properties, low density, low coefficient of thermal expansion, and high thermal conductivity [33]. The introduction of CNF into the composite materials improves the performance properties of the product and also improves the technological properties of the original composite powder material. Thus, CNFs are a promising modifying additive for materials used in new production technologies [34]. CNFs are already widely used to improve the mechanical characteristics of building materials and can be used in the energy and biomedical industries [35,36].

Based on the above, this study aims to synthesize coatings/solid objects from aluminum–alumina–CNF composite material using the cold spray method. In previous studies, only binary composites (aluminum–carbon nanomaterial or alumina–carbon nanomaterial) were used, while the present study considers a ternary system in the range of CNF concentrations of 0.5–1.5%. It also should be noted that both the original aluminum powder and the substrate were made from the same alloy. Therefore, the present study is of high practical importance since the considered compositions of composites and synthesis regimes can be used to increase the surface properties of a particular aluminum alloy.

## 2. Materials and Methods

The initial Al powder ASP-30 (produced by UC RUSAL, Moscow, Russia) was used in this work. This powder was obtained by the molten metal spraying method and had a particle size D50 = 30 μm (maximum particle size for 50% of the cumulative mass). For the production of these powders, technical-grade aluminum (99.7% Al) was used. Particle size distribution was determined using the laser diffraction method on the Analysette 22 NanoTecPlus device (Fritsch GmbH, Idar-Oberstein, Germany) with a full-scale range of 0.01–2000 µm. Al_2_O_3_ was obtained from aluminum chloride [37] that consisted of no more than 1% of impurities and corresponded to the α-modification of alumina. It was processed in a ball mill and thoroughly sieved. The average size of the Al_2_O_3_ powder was D50 = 20 µm. CNF was prepared by the CVD method. Conditions for CNFs production used in the study were the following: T = 650 °C in propane-butane on Ni–Cu catalyst. The particle size of the CNFs used did not exceed 600 nm. A detailed description of the production method and characterization of the CNFs used are presented in the study [38].

Powder morphology and microstructure characterization were performed using scanning electron microscopy (SEM) with FEI Quanta 200 (Hillsboro, OR, USA). Chemical composition and maps of the chemical element distribution in the material were obtained using an X-ray energy dispersive microanalysis system (EDS), Octane Super EDS. CNF morphology was studied using a JEM-2100 transmission electron microscope (JEOL Ltd., Tokyo, Japan). The morphology and microstructure of the obtained samples were controlled by optical microscopy using a Carl Zeiss Axio Observer A1m (Oberkochen, Germany) microscope. For microstructure analysis and porosity measurement, ImageExpert Pro 3 software was used. The etching for microstructural investigations was performed in Keller’s reagent. Microhardness was measured using Tukon 1102 (ITW Test & Measurement GmbH, Dusseldorf, Germany) with an applied load of 50 g, 10 sec exposure, and ten measurements for each sample. X-ray diffraction analysis (XRD) was carried out on a Rigaku Ultima IV (Tokyo, Japan) X-ray diffractometer using CuKα radiation. Four sample compositions (see Table 1) were prepared for cold spray (CS). The weight of every prepared powder sample was 240 g.

**Figure 1:**
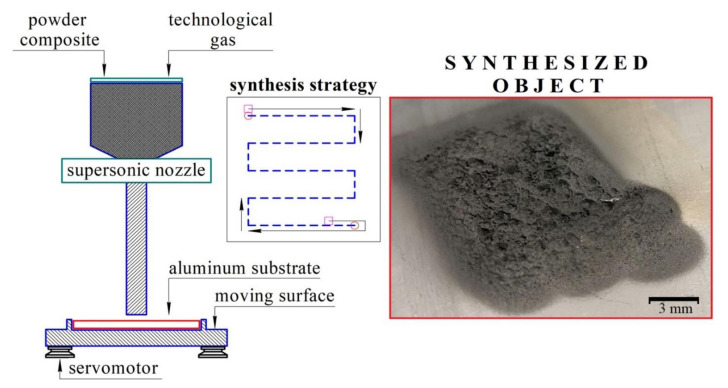
DIMET cold spray equipment.

Powders were mixed in a planetary mill using an argon atmosphere and vacuum system to prevent mechanoactivation. Steel balls, 4 g each, were used as mixing bodies. The powder-to-steel balls mass ratio was 1:20. The mixing procedure consisted of three iterations of 3 min of mixing and a 1 min break between iterations. After mixing prepared powders were thoroughly sieved. CS of the prepared powder mixtures was made using DIMET equipment shown in Figure 1.

The powder was sprayed at 7-bar pressure and, after heating to 400 °C, it was deposited on the AlSi12 aluminum alloy substrate. The speed of nozzle movement was 1 mm/s. The size of each sample was 12 × 12 mm^2^, with at least 1 mm deposited layer thickness.

## 3. Results and Discussion

**Figure 2:**
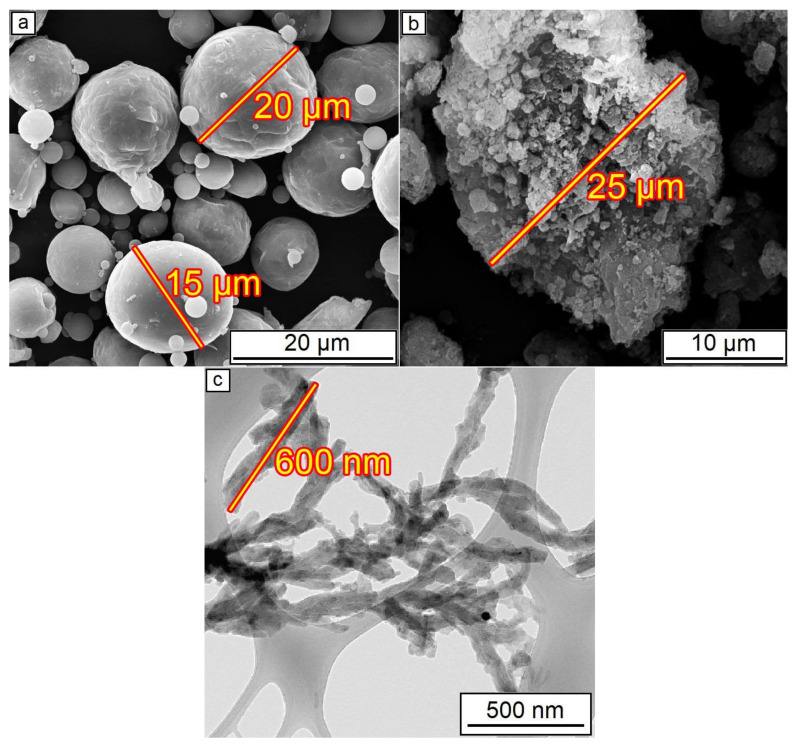
Initial powders before mixing: (**a**) Al powder; (**b**) Al_2_O_3_ powder; (**c**) CNF.

The SEM of initial powders is presented in Figure 2. The initial Al_2_O_3_ powder consisted of agglomerates with a 25 µm diameter. During mixing in a mill, these agglomerates were ground to the more dispersed particles with approximately 2–3 µm diameter. A 25–35 µm size order of both aluminum and alumina particles/agglomerations was shown [39] as optimal to form dense coating during CS.

The presence of around 30 wt.% of aluminum on the coating composition is essential since pure Al_2_O_3_ powder, which consists of hard particles, cannot be used for cold spraying without ductile additions. Hard ceramic particles could only form a monolayer on the ductile aluminum surface, whereas further layers do not have enough adhesion to continue coating formation [39]. This reason also leads to a decrease in the deposition efficiency, i.e., the volume of alumina in the coating is always less than in the initial mixture [40]. The estimated deposition efficiency of the CS of Al–Al_2_O_3_ mixture with 70 wt.% of alumina is around 10–15% which is similar to the efficiency of pure Al cold spraying, wherein alumina content in the coating is about two times lower than in the feedstock powder [22]. The agglomerations of alumina observed in Figure 2b also could influence the loss phenomena since the destruction of such agglomerates due to collisions during the CS process possibly leads to random scattering of their components. All these features of Al_2_O_3_ cold spraying should be taken into account during the planning of experiments and analysis of the coatings’ microstructure.

**Figure 3:**
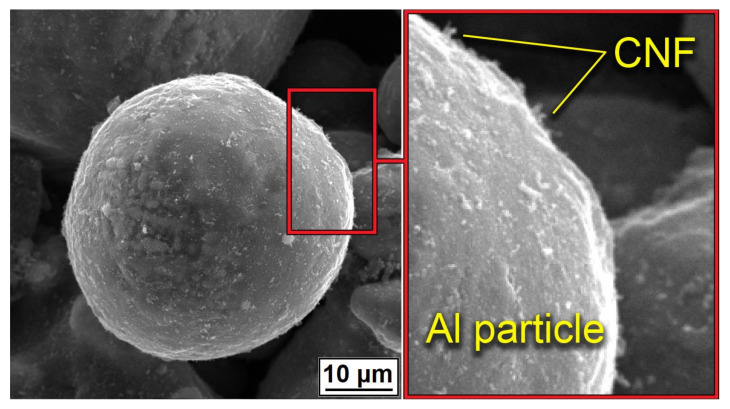
Distribution of CNF on the surface of Al powder after mixing.

Figure 3 demonstrated the distribution of CNF on the surface of Al powder after mixing. Powders demonstrated a homogeneous covering of particles with CNF without any noticeable agglomerations. Along with the homogeneous distribution of CNFs, the particle shape and morphology of the aluminum were almost unchanged.

**Figure 4:**
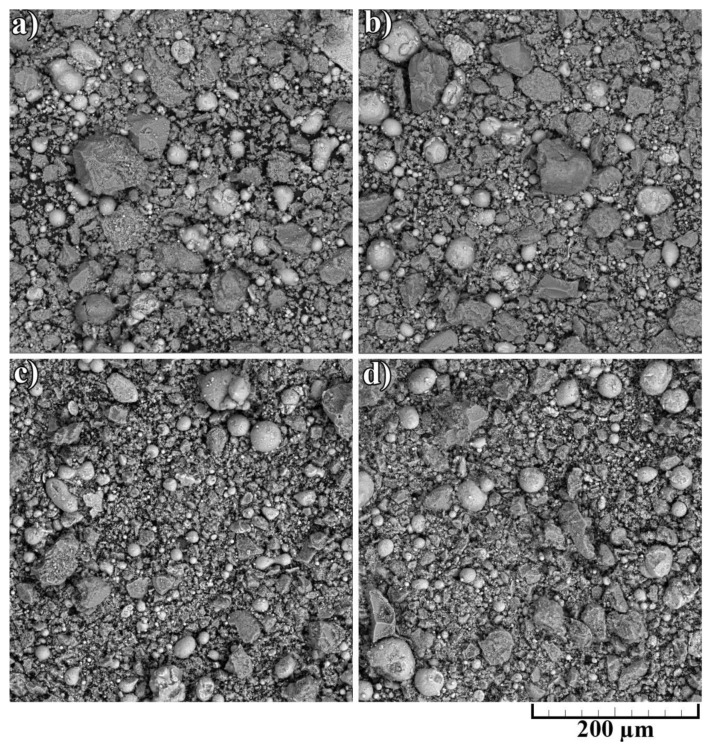
Morphology of composite powders after mixing: (**a**) 30% Al + 70% Al_2_O_3_; (**b**) 29.5% Al + 70% Al_2_O_3_ + 0.5% CNF; (**c**) 29% Al + 70% Al_2_O_3_ + 1.0% CNF; (**d**) 28.5% Al + 70% Al_2_O_3_ + 1.5% CNF.

The noticeable transformation occurred in the CNF length, which significantly decreased. Since nano-sized carbon matter possesses high strength along with high brittleness, the mixing procedure led to the destruction of the CNFs. On the one hand, short fibers concede the long ones in strengthening effect; on the other hand, it is simpler to obtain a homogeneous distribution of CNFs on the aluminum particles’ surface. The overall morphology of composite powders after mixing at low magnification is shown in Figure 4. The presented SEM results demonstrated the efficiency of the powder mixing process. For an efficient CS process, the high quality of Al_2_O_3_ and CNF distribution is essential to obtain a dense and defect-free layer with increased mechanical properties.

**Figure 5:**
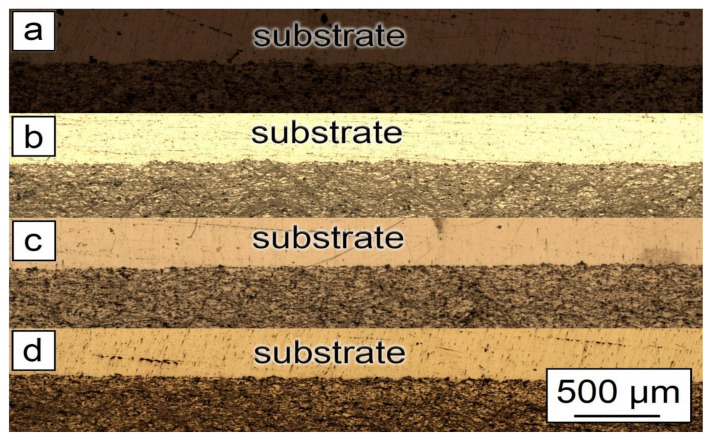
Morphology of samples after CSM: (**a**) 30% Al + 70% Al_2_O_3_; (**b**) 29.5% Al + 70% Al_2_O_3_ + 0.5% CNF; (**c**) 29% Al + 70% Al_2_O_3_ + 1.0% CNF; (**d**) 28.5% Al + 70% Al_2_O_3_ + 1.5% CNF.

**Figure 6:**
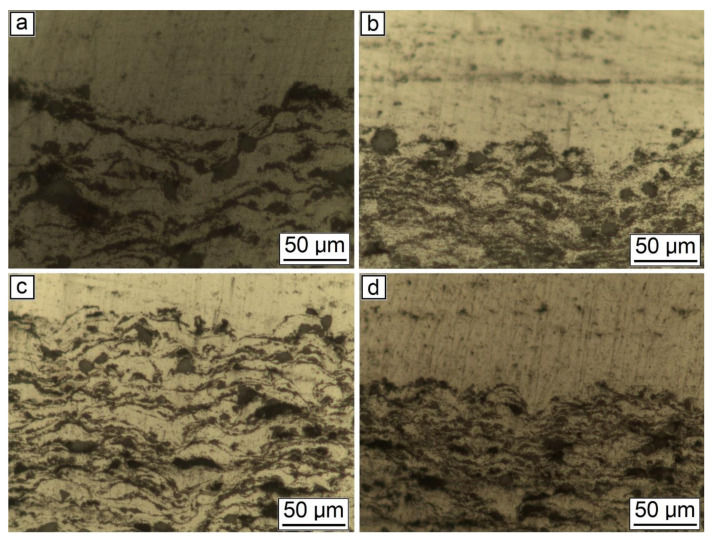
Microstructure of the synthesized samples: (**a**) 30% Al + 70% Al_2_O_3_; (**b**) 29.5% Al + 70% Al_2_O_3_ + 0.5% CNF; (**c**) 29% Al + 70% Al_2_O_3_ + 1.0% CNF; (**d**) 28.5% Al + 70% Al_2_O_3_ + 1.5% CNF.

Figure 5 and Figure 6 demonstrate the optical microscopy of the samples after CS.

As can be seen from Figure 5, the general features of the images were identical for all studied samples. Each sample had good adhesion to the substrate and closely adhered to the construction plane. The microstructure of the synthesized samples is shown in Figure 6. As can be seen from the figure, each sample had a gradient structure, and with an increase in the CNF amount, the frequency of the construction layers interchange increased. The dark areas on the figures represent a powder material with a high content of alumina (Al_2_O_3_). In Figure 6a, the distance between layers was about 15 µm, but in Figure 6d, there was practically no distance between the dark areas, and the layers were more densely packed. Additionally, a decrease in the number of agglomerated particles with an increase in the amount of CNF was noticed. As can be seen from Figure 6, with an increase in CNF concentration, the number of agglomerates decreased significantly. Figure 6a,b demonstrated spheroidal-shaped agglomerates. The size of such agglomerates decreased with the increase in the CNF amount, and in the case of 1.5% of CNF (Figure 6d), almost no agglomerations were noticed.

**Figure 7:**
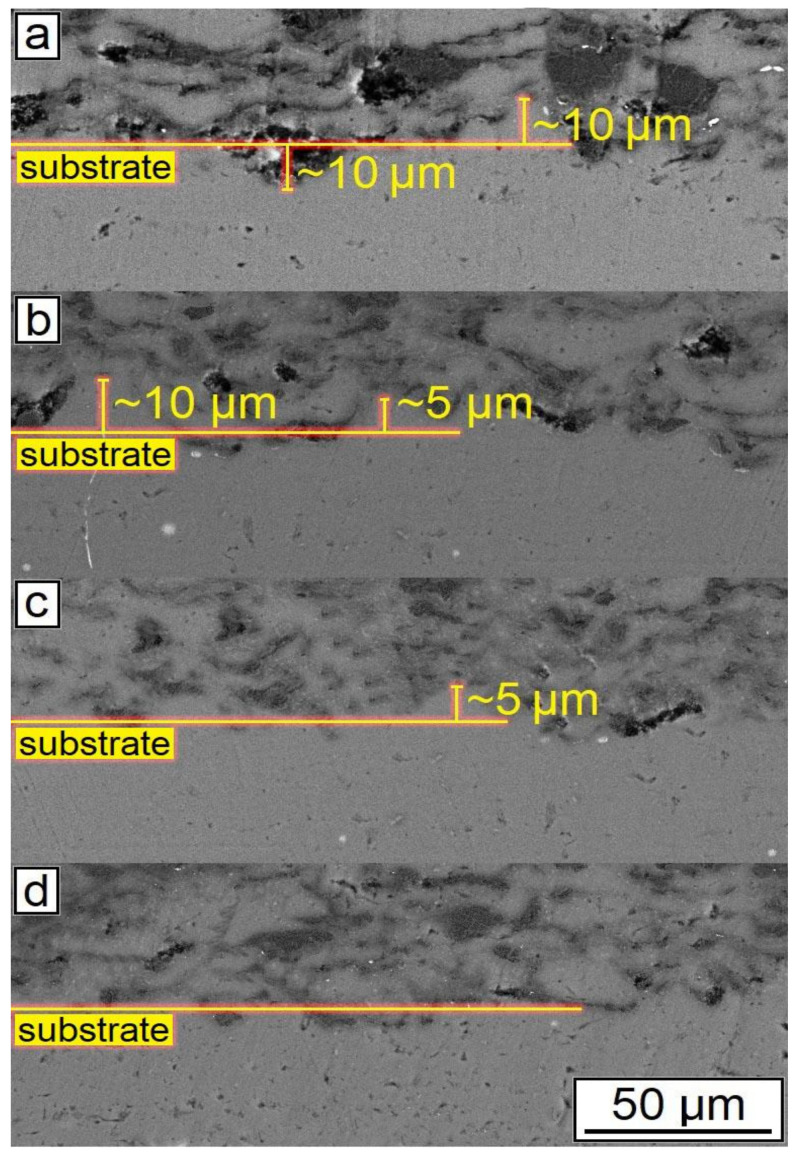
SEM results of the synthesized samples: (**a**) 30% Al + 70% Al_2_O_3_; (**b**) 29.5% Al + 70% Al_2_O_3_ + 0.5% CNF; (**c**) 29% Al + 70% Al_2_O_3_ + 1.0% CNF; (**d**) 28.5% Al + 70% Al_2_O_3_ + 1.5% CNF.

**Figure 8:**
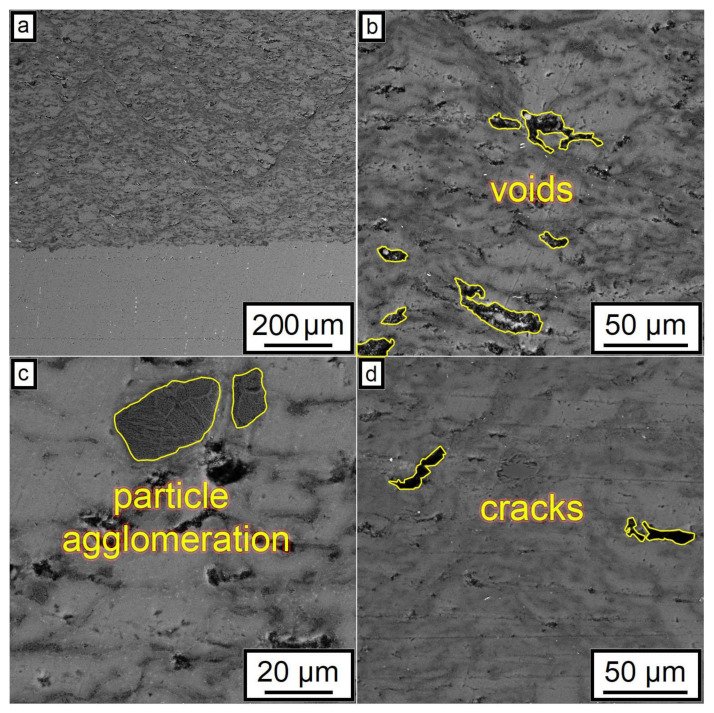
Typical defects of microstructure after CSM demonstrated using the 29% Al + 70% Al_2_O_3_ + 1.0% CNF sample: (**a**) microstructure overview; (**b**) example of voids within coating microstructure; (**c**) example of particle agglomerations; (**d**) cracks.

The SEM results shown in Figure 7 also indicated that an increase in the CNF amount has a positive effect on the density of the synthesized samples. According to the obtained microstructures, samples containing 1.0–1.5% of CNF have noticeably fewer microstructural defects compared to the samples with 0.5% of CNF. The porosity for the samples with 0.5%, 1%, and 1.5%, CNF content were found to be 4.6 ± 0.5%, 3.1 ± 0.9%, and 2.0 ± 0.5%, respectively. Ductile Al particles promote defect-free structure formation by deforming plastically when interacting with brittle and hard Al_2_O_3_ particles and CNF during cold spray. On the other side, ceramic particles are unable to provide plastic deformation when interacting with each other. Thus, the main source of the obtained porosity is the absence of plastic deformation during the interaction between Al_2_O_3_ particles and CNF, leading to localized agglomerations, as shown in Figure 8c. Such locations of entrapped agglomerations in the structure between Al regions are the main type of porosity obtained in the samples.

The change in the phase interface also indicated the influence of the amount of CNF on the formation of the surface layer (see Figure 7). As can be seen, with the highest content of CNF, the boundary line is smooth, and with a smaller amount of CNF, the phase interface has a wave character. This is probably due to the interaction of particles in the synthesis process. Alumina performs the function of abrasive material. When applying a layer, alumina peels the oxide film from the surface of the substrate, while the oxide layer on aluminum particles is also destroyed. CNF envelops aluminum particles due to their small size and prevents the formation of agglomerates (see Figure 8). As can be seen from Figure 7 and Figure 8, with the largest amount of CNF, the synthesized sample has the least number of defects.

**Figure 9:**
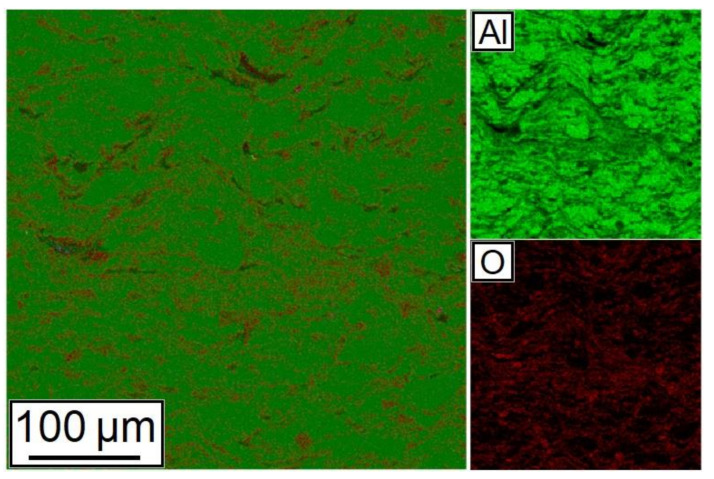
Distribution of chemical elements in the cross-section of the 28.5% Al + 70% Al_2_O_3_ + 1.5% CNF sample.

A map of chemical elements’ distribution is shown in Figure 9. As can be seen from the figure, Al and Al_2_O_3_ particles have a good distribution in the synthesized sample.

**Figure 10:**
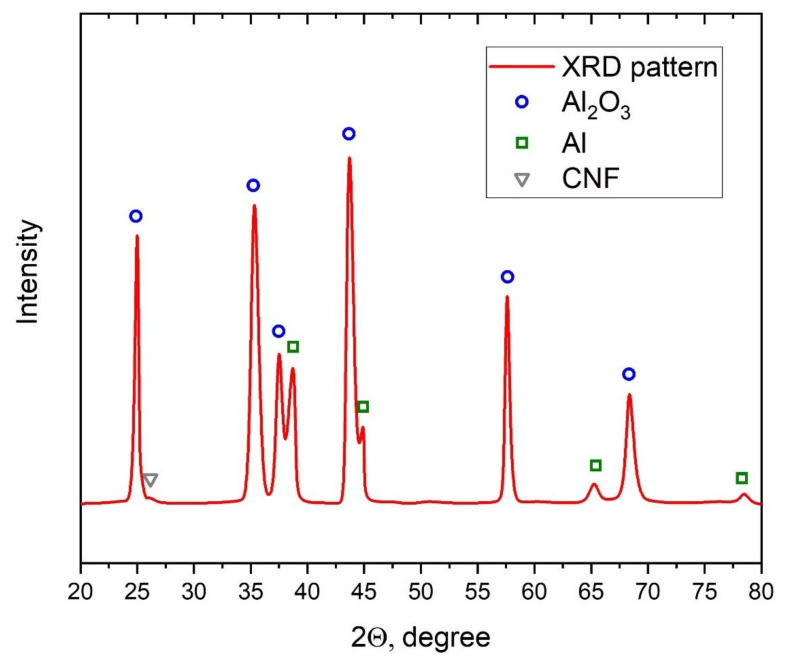
XRD of the 28.5% Al + 70% Al_2_O_3_ + 1.5% CNF sample.

To control the presence of CNF in the sample after the cold spray process, the XRD analysis of the sample (see Figure 10) with 1.5% CNF addition was performed. The synthesized sample was detached from the substrate to ensure the analysis results. Based on the XRD results, besides characteristic peaks for Al and Al_2_O_3_, a small characteristic peak of CNF at around 26 degrees was obtained. This peak demonstrated the presence of CNF in the structure of composite after cold spray synthesis.

Based on the obtained results, CNF had a positive effect on the structure of the synthesized samples. The obtained effect was associated with the following factors. CNF increases the bulk density of the initial powder material due to the smaller size compared to the initial Al powder. Due to such size differences, voids between the larger powder particles filled with smaller CNF, increasing the density of the synthesized samples. The second factor is the high antifriction properties of carbon. CNF reduces the number of agglomerates in the initial powder, resulting in a decrease in agglomerated particles in the synthesized samples. The described factors have a positive effect on the overall microstructure of the synthesized samples, reducing the number of cracks and voids.

**Figure 11:**
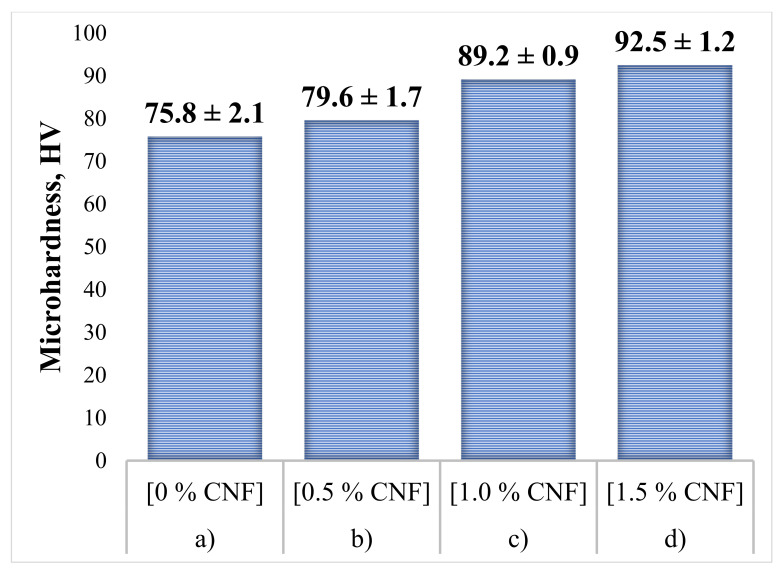
Microhardness of the synthesized samples: (**a**) 30% Al + 70% Al_2_O_3_; (**b**) 29.5% Al + 70% Al_2_O_3_ + 0.5% CNF; (**c**) 29% Al + 70% Al_2_O_3_ + 1.0% CNF; (**d**) 28.5% Al + 70% Al_2_O_3_ + 1.5% CNF.

To determine the effect of CNF content on the mechanical properties of the specimens, hardness tests were carried out. As a comparison, the average microhardness value for the initial Al powder without additions of Al_2_O_3_ and CNF is 24.1 ± 1.2 HV. The obtained microhardness results, measured in the cross-section of the synthesized samples, are presented in Figure 11. For each sample, 15 measurements were carried out. Following the obtained results, it can be concluded that the hardness level of the samples increases with an increase in CNF content. The obtained hardness increase in the case of 1.5% CNF addition is about 20% and was associated with three main contributions. The first one is the dispersion strengthening mechanism due to the much higher strength and hardness of alumina compared with the aluminum matrix. Only a 20% increase in hardness could be explained by alumina loss during the CS process, as was mentioned above. The second contribution concerns the mechanical deformation of the ductile Al particles within the coating because of collision with the substrate surface, hard alumina particles, and each other. The deformation of the particles which become grains within the coating leads to an increase in dislocation concentration and hardening of the material. The third contribution is associated with the presence of CNF with also high strength and hardness. This contribution is relatively small because of the low CNF concentrations, but their nanoscale and uniform distribution provide reasonable strengthening.

An increase in hardness is due to the synergistic effect of using microsized alumina and nanosized carbon fibers as functional strengthening additives. Due to the good antifriction properties of carbon, the fluidity of the material increased by reducing the friction force between the particles. The nanosized additive was evenly distributed between the particles of aluminum and alumina, filling the voids and increasing the bulk density of the original composite. Additionally, due to the high thermal conductivity of carbon, the temperature gradient between the deposited layers decreased, which in turn reduced residual stresses and minimized the negative thermal effects. During the synthesis of objects by the cold spray method, nanosized CNF formed a composite structure that prevented the movement of the dislocations under mechanical loads due to the Orowan strengthening mechanism.

## 4. Conclusions

1. Al–Al_2_O_3_–CNF powder composite materials for the cold spray process are investigated in this study. The method of preparation of powder composites and the process of sample synthesis are considered in detail.
2. CNF affects the microstructure of samples synthesized by the cold spray method. This is due to an increase in the bulk density of the initial powder material, as well as the high antifriction properties of carbon, which significantly affects the density of samples and reduces the number of agglomerates.
3. Increasing the concentration of CNF has a positive effect on the hardness of synthesized objects. With a CNF content of 1.5%, the microhardness of the samples is on average 20% higher, which is due to the mechanism of dispersive strengthening.

## Figures and Tables

**Table 1:** Powder samples compositions.

Sample	Component Content, wt.%		
	Al	Al_2_O_3_	CNF
1	30	70	-
2	29.5	70	0.5
3	29	70	1.0
4	28.5	70	1.5

## Data Availability

The data presented in this study are available on request from the corresponding author. The data are not publicly available due to privacy.
